# Should we abandon the simple aspiration in favour of chest tube drainage in the management of spontaneous pneumothorax? A systematic review and meta-analysis

**DOI:** 10.1186/s12890-026-04447-0

**Published:** 2026-06-25

**Authors:** Tom Caput, César Guerrin, Anastasia Boitel, François Calais, Frédéric Mauny, Tania Marx

**Affiliations:** 1 Laboratoire chrono-environnement 6249 CNRS, Université Marie et Louis Pasteur, 32 avenue de l’Observatoire, Besançon, 25000 France; 2 Département de médecine d’urgence, CHU de Besançon, Besançon, F-25000 France; 3 Inserm CIC-uMETH 1431 centre hospitalier universitaire de Besançon, Besançon, France; 4 Université Marie et Louis pasteur, 32 avenue de l’Observatoire, Besançon, 25000 France

**Keywords:** Meta-analysis, Systematic review, Simple aspiration, Spontaneous pneumothorax, Chest tube drainage

## Abstract

**Backgrounds:**

Following the latest studies, the choice between chest tube drainage and simple aspiration remains debated in the treatment of spontaneous pneumothorax. The aim of this study was to compare the efficacy between SA and CTD in treatment of spontaneous pneumothorax in adults in a systematic review and meta-analysis.

**Methods:**

Literature research was conducted with Medline, Embase and Central databases from inception to march 2025. The target population is adults presenting a SP managed by SA or CTD among randomized control trials and retrospective comparative studies. The RoB-2 and NOS was carried out for the quality assessment of studies. A meta-analysis was conducted with inverse variance and a random effect model.

**Results:**

19 studies were included in the meta-analysis. The results of meta-analysis comparing SA vs. CTD were as follows: immediate success OR = 0.42 (95% CI= [0.16, 1.09], *p* = 0.07), adverse effect occurrence OR = 0.22 (95% CI=[0.08, 0.61], *p* = 0.004), length of hospitalization MD = -2.59 (95% CI=[-3.57, -1.61], *p* < 0.001), pain score MD=-1.34 (95% CI=[-1.74, -0.95], *p* < 0.001).

**Conclusion:**

This meta-analysis shows no difference in the efficacy between SA and CTD in the treatment of SP, especially for PSP. However, the additional benefits of SA over CTD in clinical patient management support prioritizing this strategy, in line with international guideline recommendations.

**Supplementary Information:**

The online version contains supplementary material available at https://doi.org/10.1186/s12890-026-04447-0.

## Introduction

Spontaneous pneumothorax (SP) is defined by the presence of air in the pleural space that occurs without traumatic factors. Two categories are available: primary spontaneous pneumothorax (PSP) occurring in healthy lungs and secondary spontaneous pneumothorax (SSP) with the presence of a pulmonary disease [1]. In United Kingdom in 2016, the annual incidence was to 14.1 per 100 000 with hospital admissions on the rise since 1968 and the mortality rate in 2000 was 1.26 per million per year for men and 0.62 per million per year for women [2, 3]. In 1997, Baumann and al. considered the annual costs in the United States around 130 million dollars [4].

According to the British Thoracic Society Guideline updated in 2023, patient management is driven by different factors. When SP is asymptomatic or minimally symptomatic, the patient should only be monitored without any intervention. In case of symptomatic pneumothorax or high-risk characteristics, interventional treatment is required with chest tube drainage (CTD) or simple aspiration (SA) [5].

The choice of treatment between CTD or SA is currently debated. Between 2017 and 2023, five recent meta-analyses were conducted with different conclusions. Vuong et al., Tan et al., Mummadi et al. and Eamer et al. did not reveal any significant differences but Carson-Chahhoud et al. concluded in favour of CTD [6–10]. In 2023, a major non-inferiority trial conducted by Marx and al. demonstrate a higher failure rate for SA compared to CTD but with a better tolerance [11]. After this publication, a new meta-analysis conducted by Cheng et al. was published [12]. This study presents CTD as more effective than SA in contradiction with the last guidelines [5, 13]. Nevertheless, some methodological considerations can be debated in this study which reduces the clinical and practical value of the results: 1/ the only consideration of randomized control trial studies without even taking into account large-scale, high-quality observational studies and 2/ not making the distinction between immediate success, 24 h success and 7 days success independently. With the aim of helping practitioners in their choice of treatment, we suggest conducting a new systematic review and meta-analysis with the aim of comparing the clinical efficacy between CTD and SA in the treatment of SP.

## Method

This study is based on the PRISMA (Preferred Reporting Items for Systematic Reviews and Meta-analyses) guidelines [14]. The Cochrane Collaboration Handbook for Systematic Reviews of Interventions and Meta-analysis of Observational Studies in Epidemiology (MOOSE) was used to carry out the study [15, 16]. The systematic review was registered in the “International prospective register of systematic reviews (PROSPERO)” with the registration number “CRD42023482759” on 24/11/2023.

### Search strategy

The literature research was conducted with Medline, Embase and Central (Cochrane Central Register of Controlled Trials) databases from inception to March 2025, to find scientific research articles evaluating the efficacy of CTD and SA for the treatment of SP. Data sources were journal articles for published data and clinical study reports or available data for studies in progress.

Searches for unpublished studies have been conducted considering randomized controlled trials in instance at ClinicalTrials.gov and EudraCT clinical trial records over the same period. Keywords used for request have been based on the following words and adjusted on databases: “pneumothorax”, “treatment”, “thoracic tube drainage”, “exsufflation” (supplementary data Table 1). Bibliographic searches were conducted by a professional librarian (F.C.), Université Marie et Louis Pasteur, France. Additional publications were identified by screening the references in the bibliography of the included studies and previous systematic reviews and meta-analysis.

### Study selection

We included studies that described the efficacy of CTD compared to SA in the management of SP, based on the PICOS method (supplementary data Table 2). The target population was adults (> 18 years) having presented a SP and having been managed by CTD or SA. We included randomized control trials and observational studies (cross-sectional, prospective, or retrospective research design, case-control) written in English or French that have compared CTD and SA in treatment of SP. According to the Cochrane handbook, we don’t include other languages which will not influence the results [15].

The first outcomes were the immediate efficacy, in 24 h and in seven days of the interventions. The success was defined individually by each methodology study. Second outcomes were the recurrence rate in one year, the number and type of adverse effect (mortality, and other adverse effects), the tolerance of treatment (dyspnea and thoracic pain scores) and the length of hospital stay.

For the meta-analysis, we included articles with a quantitative analysis of the effect of intervention for at least one of the outcomes. A comparative approach was not necessary for observational study. The case series or studies assessing only one procedure were excluded from the systematic review and analysis. Studies were independently selected by two reviewers (T.C. and C.G.) after removing duplicates. The conflicts were resolved by consensus with a third author (T.M.). This part was managed by RAYYAN ©.

### Data extraction and quality assessment

The following data were extracted from each study: 1] article’s information (title’s article, author, year of publication, and location of study) 2] study design (type of study and type of analysis for RCT) 3] population (age means, sex ratio, tobacco or cannabis ratio, type of SP, number of pre-existing diseases for SSP, number of subjects in each group of treatment, number of centre) 4] outcomes. Review Manager (RevMan©) was used for recording decisions. When necessary, the article’s author was contacted by mail to collect data to exploit the results from current randomized control trials and studies not available in their entirety. Two reviewers (T.C. and C.G.) extracted data independently. Conflict was managed between them with consensus.

The quality assessment of the randomized control trials has been carried out by the Cochrane risk of bias tool for randomized trials (RoB 2) and the non-randomized study by the Newcastle-Ottawa Scale (NOS) [15, 17]. The first scale checks five potential risks of bias sources (randomization process, deviations from intended interventions, missing outcome data, measurement of the outcome and selection of the reported result). The final decision for studies was “Low”, “High” or “Some concerns” risk of bias. The NOS is based on other items (selection, comparability, exposure and outcome) and is rated on a point scale from zero (poor) to nine (excellent) [18]. Based on Zhang et al. assessment, we categorized the studies as “high” (0–3), “moderate” (4–6) or “low” (7–9) risk of bias [19].

### Statistics analysis

Quantitative data was extracted with 2 × 2 tables for each dichotomous outcome and means, standard error and population for continuous outcomes. For RCT, data from intention to treat and per protocol analysis were both extracted.

Continuous data reported in articles with median and quartiles were transformed into median and standard deviation according to formulas presented by Wan et al. [20]. Odds ratios (OR) with confidence interval (CI) were calculated for the main outcome and additional outcome (adverse effect and recurrence) and mean difference (MD) for the duration of hospitalization and tolerance of treatment has been also calculated. Inverse variance model with a random effect model (DerSimonian and Laird method) was implemented. Heterogeneity was assessed using: (i) the Chi-squared test included in the forest plots (at a significance threshold set to 0.05) and (ii) the I² statistic. Heterogeneity among studies was classified as low (I²< 25%), moderate (25 < I² <75%), or high (I²> 75%). We conducted a heterogeneity exploration based on subgroup analysis, in addition to a random effect modelling conducted on the following criteria: the quality studies, the type of population (PSP or SSP) and the study design (randomized controlled trial study versus observational study). Funnel plots were exploited to represent the measure of the study precision (standard error) versus the effect size of each study. A roughly symmetrical funnel plot indicates very low (or no) publication bias. Review Manager (RevMan) (The Cochrane Collaboration, 2014) was used to conduct the analysis.

## Results

**Fig. 1:**
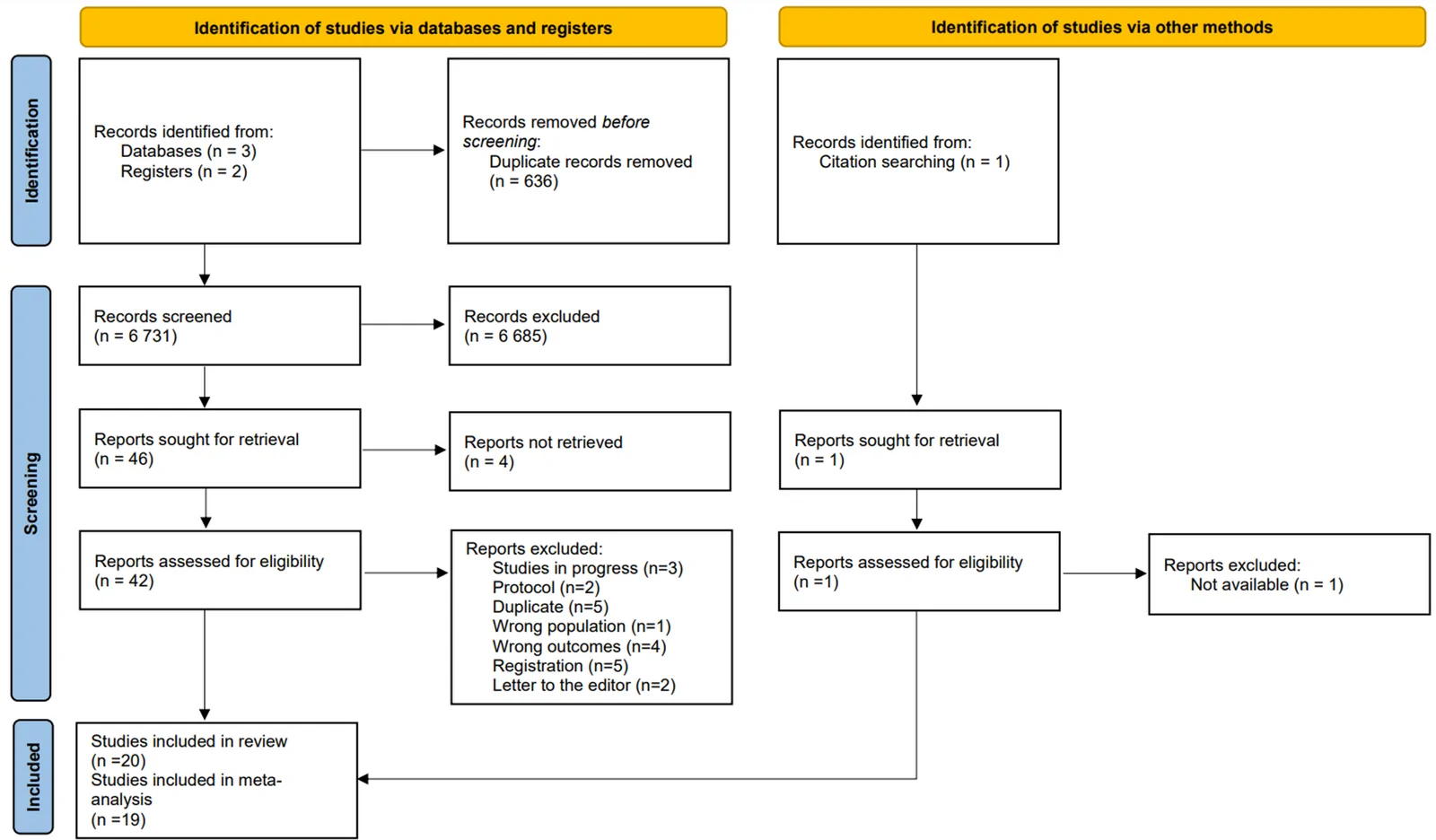
PRISMA flow diagram

After a first removal of duplicates, 6 731 articles were identified in the systematic research. Then, with reading title and abstract, 6 685 articles have been excluded. Among the remaining 46 articles, 20 were included in the systematic review after reading the full text (Fig. 1). Four articles were not available in their entirety [21–24]. Authors were contacted to request a copy. We have received either no reply or negative reply. The hand search with the reference included articles has enabled us to identify one article [25]. It was not available, and the author has not replied to our requests. Therefore, these five studies are not included in our analysis.

### Characteristics of included studies

The publication period ranged from 1963 to 2023. Eight studies have been conducted in Europe [26–32], six in Asia [33–38], three in the Middle East [39–41], one in North Africa [42], one in Australia [43] and one in North America [44]. Eleven were RCTs including one non inferiority trial. Nine were observational studies including one prospective and eight retrospectives’ studies. Ten articles studied only PSP, none studied only SSP and ten studied all SP. Description of included articles is presented in two Tables (Tables 1 and 2).

**Table 1:** Characteristics of included RCTs

Author, year	Location of study	Study period	Study design	Population, Simple size	Inclusion of pneumothorax recurrence	Inclusion of tension pneumothorax	number of attempts to SA	measured outcomes	Risk of bias
T, MARX 2023 [11]	France	2009–2015	RCT Non-inferiority multicentric	PSP *N* = 379	No	No	2 attempts	Success at 24 h Success at 7 days Global pain Dyspnea Occurrence of adverse event Recurrence at 1 year	Some concerns
I.H, KIM 2019 [36]	Korea	2015–2016	RCT Superiority monocentric	PSP *N* = 40	No	No	2 attempts	Success at 24 h Recurrence at 1 year Length of hospitalization	Some concerns
A, RAMOUZ 2018 [40]	Iran	2017–2018	RCT Superiority multicentric	PSP *N* = 70	NI	No	3 attempts	Immediate success Success at 7 days Global pain Occurrence of adverse event Recurrence at 1 year Length of hospitalization	Some concerns
A, THELLE 2017 [31]	Norway	NI	RCT Superiority multicentric	PSP *N* = 79 SSP *N* = 48	Yes	No	3 attempts	Immediate success Success at 7 days Occurrence of adverse event Length of hospitalization	Some concerns
P, KORCZYNSKI 2015 [32]	Poland	2009–2014	RCT Superiority monocentric	PSP *N* = 34 SSP *N* = 15	Yes	No	2 attempts	Success at 7 days Length of hospitalization	High
K.K, HO 2011 [37]	Singapore	2004–2006	RCT Superiority monocentric	PSP *N* = 48	Yes	No	1 attempt	Immediate success Global pain Occurrence of adverse event Length of hospitalization	Low
I, MASOOD 2007 [38]	India	NA	RCT Superiority NI	All SP *N* = 82	No	No	1 attempt	Success at 24 h Occurrence of adverse event Length of hospitalization	High
A.K, AYED 2006 [41]	Kuwait	2001–2003	RCT Superiority monocentric	PSP *N* = 137	No	No	2 attempts	Immediate success Success at 7 days Global pain Occurrence of adverse event Recurrence at 1 year Length of hospitalization	High
M, NOPPEN 2002 [28]	Belgium	NA	RCT Superiority multicentric	PSP *N* = 60	No	No	2 attempts	Immediate success Success at 7 days Recurrence at 1 year Length of hospitalization	High
P, ANDRIVET 1995 [29]	France	NA	RCT Superiority multicentric	PSP *N* = 53 SSP *N* = 8	No	Yes	2 attempts	Success at 24 h Global pain Dyspnea Occurrence of adverse event Length of hospitalization	High
J, HARVEY 1994 [30]	Scotland	NA	RCT Superiority NI	PSP *N* = 73	No	No	2 attempts	Immediate success Global pain Recurrence at 1 year Length of hospitalization	High

*NA* not applicable, *PSP* primary spontaneous pneumothorax, *SSP* secondary spontaneous pneumothorax

**Table 2:** Characteristics of included observational studies

Author, year	Location of study	Study period	Study design	Population, Simple size	Inclusion of pneumothorax recurrence	Inclusion of tension pneumothorax	number of attempts to SA	measured outcomes	Risk of bias
T, Nakano 2023 [33]	Japan	2006–2015	Observational Retrospective Monocentric	PSP *N* = 165 SSP *N* = 305	Yes	No	2 attempts	Immediate success Occurrence of adverse event Recurrence at 1 year	Low
B, Tasneem 2022 [39]	Pakistan	2021–2022	Observational Prospective Monocentric	PSP *N* = 58	NA	Yes	1 attempt	Immediate success Success at 7 days Occurrence of adverse event Recurrence at 1 year Length of hospitalization	Low
MB, Ganaie 2018 [26]	United Kingdom	2008–2015	Observational Retrospective Monocentric	PSP *N* = 69	Yes	No	3 attempts	Immediate success Recurrence at 1 year Length of hospitalization	Low
H, Moubachir 2016 [42]	Morocco	2012–2014	Observational Retrospective Monocentric	PSP *N* = 17 SSP *N* = 116	No	No	1 attempt	Immediate success Occurrence of adverse event Length of hospitalization	Low
JW, Chan 2009 [34]	Hong Kong	2004–2004	Observational Retrospective Multicentric	PSP *N* = 400 SSP *N* = 387	Yes	NA	1 attempt	Immediate success Occurrence of adverse event Length of hospitalization	Low
AM, Kelly 2008 [43]	Australia	1996–2005	Observational Retrospective Multicentric	PSP *N* = 112	Yes	No	NA	Immediate success	Low
M, Eng Hock Ong 2004 [35]	Singapore	2000–2001	Observational Retrospective monocentric	PSP *N* = 82 SSP *N* = 21	Yes	No	NA	Immediate success Occurrence of adverse event Recurrence at 1 year Length of hospitalization	Low
JM, Vernejoux 2001 [27]	France	1988–1991	Observational Retrospective Monocentric	All SP *N* = 36	Yes	NA	NA	Immediate success Occurrence of adverse event Length of hospitalization	Low
HT.Ransdell 1963 [44]	United States	1950–1961	Observational Retrospective Multicentric	All SP *N* = 198	Yes	NA	NA	Immediate success Occurrence of adverse event Length of hospitalization	moderate

*NA* not applicable, *PSP* primary spontaneous pneumothorax, *SSP* secondary spontaneous pneumothorax

We observe a clinical heterogeneity among the included studies according to the type and size of the pneumothorax. Eight articles studied the first occurrence of SP, ten also included recurrence and two did not specify the type of SP. Seven studies included tension pneumothorax.

Nine studies don’t relate the type of catheter used for simple aspiration procedures. For the other articles, 16- or 18-gauge pleural or intravenous catheters were used in protocols studies except Marx et al. which used a thoracentesis kit. For the CTD procedure, six studies did not describe the type of catheter used. The other used 12- to 28-French large chest tubes. Ho et al. were the only authors which studied ambulatory management with 12 French catheters to Heimlich valves compared to SA [37].

Concerning the quality of RCTs, we classified one study to be “low-risk”, four to be “some concerns” and six to be “high risk” of bias from the Rob-2. Eight observational were classified as “low” and one “moderate” risk of bias with the NOS (Tables 1 and 2).

Deaths were only reported in study of Ransdell et al. [44]. The authors identified nine deaths for CTD and three for SA among samples of 95 and 103 subjects, respectively. None of the other nine studies where the mortality rate was collected identified deaths.

### Meta-analysis

Nine-teen articles were selected for the meta-analysis. No quantitative data was available for the study of Vernejoux et al., this article was not retained for the meta-analysis after data extraction [27].

#### Immediate success

**Fig. 2:**
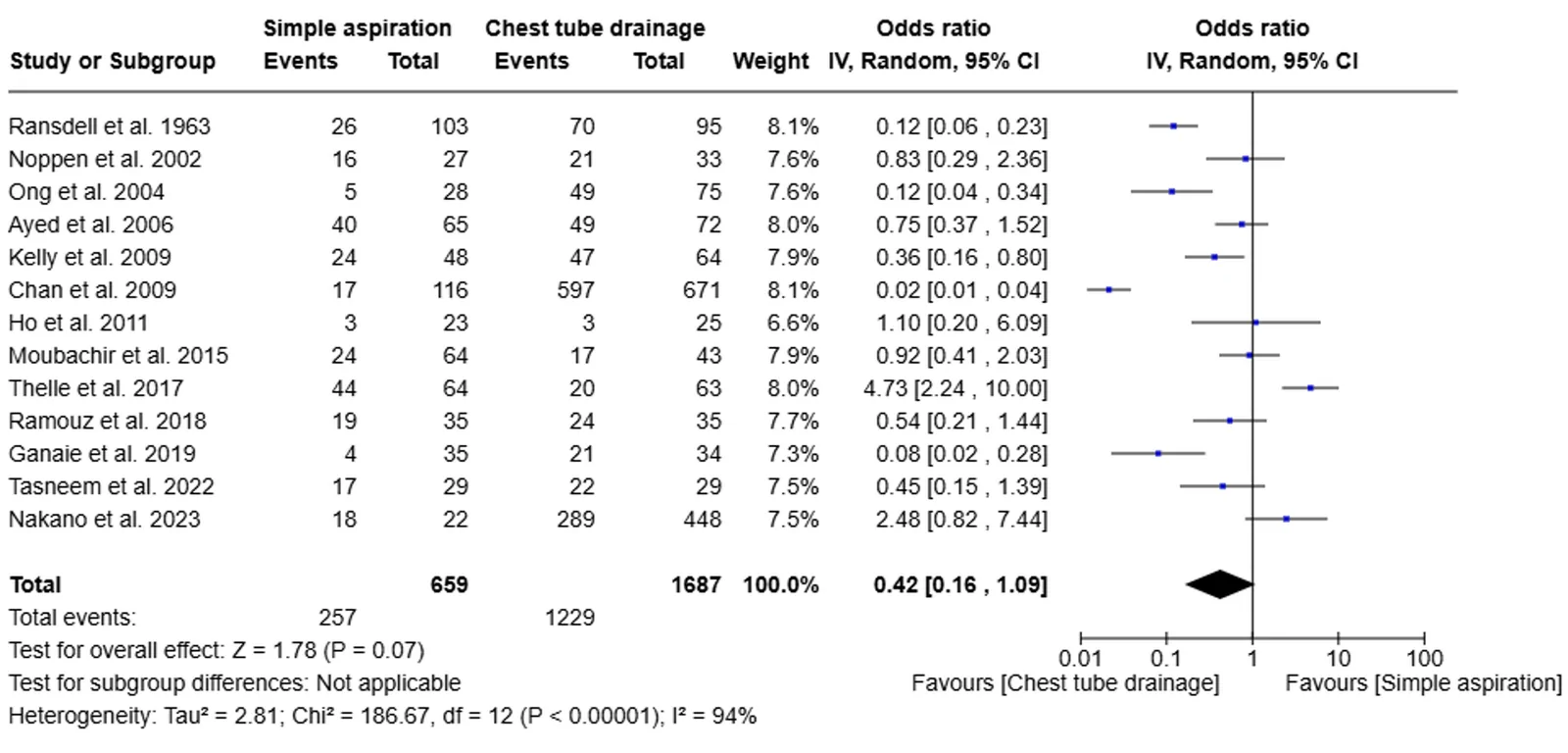
Meta-analysis of immediate success for SP between simple aspiration and chest tube drainage

The results regarding the immediate success of SA versus CTD for management of SP (PSP and SSP combined) was OR = 0.42 (95% CI= [0.16, 1.09], *p* = 0.07) (13 studies, I^2^ = 94%, *p* < 0,001) (Fig. 2. Considering only RCT studies, the results was OR = 1.15 (95% CI=[0.47, 2.81], *p* = 0.76) (five studies, I^2^ = 77%, *p* = 0.002), (supplementary data Fig. 1). The sensitivity analysis stratified on the quality of the studies yield to the same results.

The results regarding the immediate success of SA versus CTD for management of PSP was OR = 0.43 (95% CI=[0.14, 1.30], *p* = 0.14) (nine studies, I^2^ = 92%, *p* < 0.001) (supplementary data Fig. 2. Considering only RCT studies, the results was OR = 1.11 (95% CI=[0.50, 2.43], *p* = 0.80) (five studies, I^2^ = 67%, *p* = 0.02) (supplementary data Fig. 3). The sensitivity analysis stratified on the quality of the studies yield to the same results.

The results regarding the immediate success of SA versus CTD for management of SSP considering all study designs was OR = 0.33 (95% CI=[0.01, 8.03], *p* = 0.68) (three studies, I^2^ = 96, *p* < 0.001) (supplementary data Fig. 4). No sensitivity analysis on the quality of the studies was indicated in this group.

The funnel plot did not reveal any publication bias (Supplementary data Figs. 5, 6 and 7).

#### Success in 24 h

**Fig. 3:**
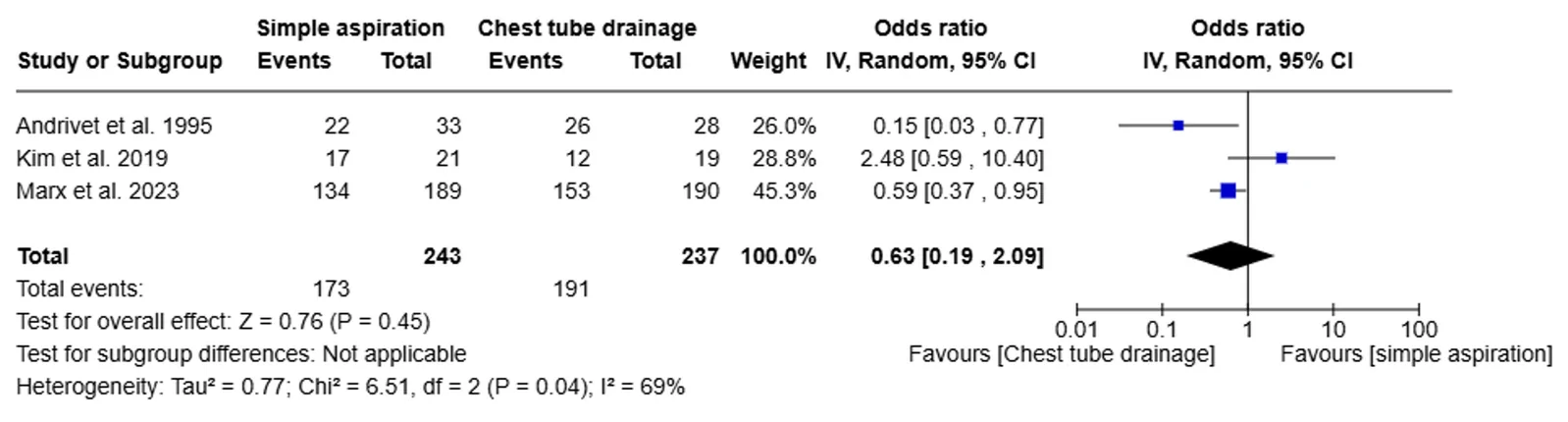
Meta-analysis of success in 24 h for SP between SA and chest tube drainage

The results regarding the success in 24 h of SA versus CTD for management of SP (PSP and SSP combined) was OR = 0.63 (95% CI=[0.19, 2.09], *p* = 0.45) (three studies, I^2^ = 69%, *p* = 0.04) (Fig. 3). The sensitivity analysis stratified on the quality of the studies yield to the same results.

The results regarding the success in 24 h of SA versus CTD for management of PSP, was OR = 1.02 (95% CI=[0.26, 4.03], *p* = 0.97) (two studies, I^2^ = 71%, *p* = 0.06) (Supplementary data Fig. 8). No sensitivity analysis on the quality of the studies was indicated in this group. Any studies have investigated the success in 24 h for SSP.

The funnel plot did not reveal any publication bias (Supplementary data Figs. 9 and 10).

#### Success in seven days

**Fig. 4:**
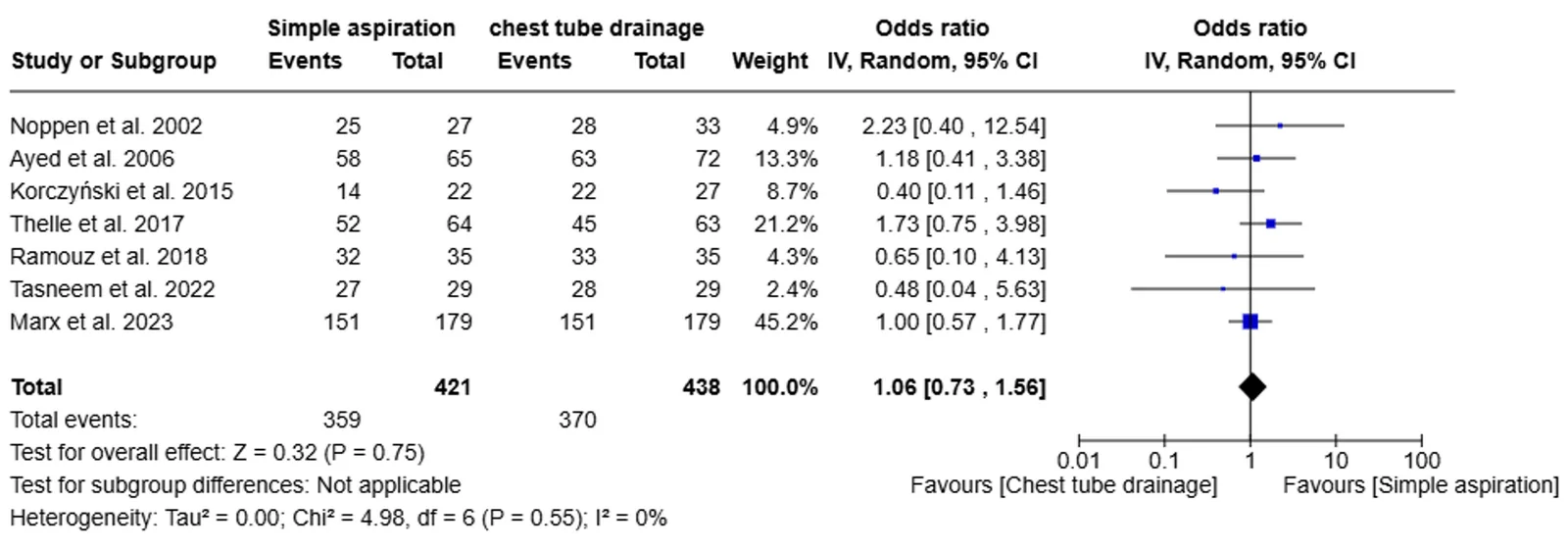
Meta-analysis of success in seven days for SP between simple aspiration and chest tube drainage

The results regarding the success in seven days of SA versus CTD for management of SP (PSP and SSP combined) was OR = 1.06 (95% CI=[0.73, 1.56], *p* = 0.75) (seven studies, I^2^ = 0%, *p* = 0.55) (Fig. 4). Considering only RCT studies, the results was OR = 1.09 (95% CI=[0.74, 1.60], *p* = 0.68) (six studies, I^2^ = 0%, *p* = 0.47) (Supplementary data Fig. 11). The sensitivity analysis stratified on the quality of the studies yield to the same results.

The results regarding the success in seven days of SA versus CTD for management of PSP was OR = 1.02 (95% CI=[0.67, 1.53], *p* = 0.94) (seven studies, I^2^ = 0%, *p* = 0.69) (Supplementary data Fig. 12). Considering only RCT studies, the results was OR = 1.04 (95% CI=[0.68, 1.57], *p* = 0.86) (six studies, I^2^ = 0%, *p* = 0.61) (Supplementary data Fig. 13). The sensitivity analysis stratified on the quality of the studies yield to the same results.

Two RCT studies investigated the success in 7 days on SSP, the results was OR = 1.45 (95% CI=[0.47, 4.50], *p* = 0.52) (two studies, I^2^ = 0%, *p* = 0.55) (Supplementary data Fig. 14). Sensitivity analysis was not indicated in this situation.

The funnel plot did not reveal any publication bias (Supplementary data Figs. 15, 16 and 17).

#### Occurrence of adverse events

The results regarding the occurrence of adverse events of SA versus CTD for management of SP (PSP and SSP combined) was OR = 0.22 (95% CI=[0.08, 0.61], *p* = 0.004) in favour of SA (six studies, I^2^ = 24%, *p* = 0.26) (Supplementary data Fig. 18). Considering only RCT studies, the results was OR = 0.21 (95% CI=[0.04, 1.15], *p* = 0.07) (four studies, I^2^ = 49%, *p* = 0.012) (Supplementary data Fig. 19).The sensitivity analysis stratified on the quality of the studies yield to the same results.

The results regarding the occurrence of adverse events of SA versus CTD for management of PSP was OR = 0.26 (95% CI=[0.08, 0.84], *p* = 0.02) in favour of SA (four studies, I^2^ = 22%, *p* = 0.28) (Supplementary data Fig. 20). Considering only RCT studies, the results was OR = 0.37 (95% CI=[0.07, 1.89], *p* = 0.23) (three studies, I^2^ = 35%, *p* = 0.21) (Supplementary data Fig. 21). The sensitivity analysis stratified on the quality of the studies yield to a non-significant result.

No studies investigated the occurrence of adverse events for SSP.

The funnel plot did not reveal any publication bias (Supplementary data Figs. 22 and 23).

#### Tolerance

The results regarding the pain score during intervention (from zero to ten) of SA versus CTD for management of PSP was MD=-1.34 (95% CI=[-1.74, -0.95], *p* < 0.001) in favour of SA (two studies, I^2^ = 0%, *p* = 0.70) (Supplementary data Fig. 24). The sensitivity analysis stratified on the quality of the studies was not necessary. The funnel plot did not reveal any publication bias (Supplementary data Fig. 25). Four other articles have assessed pain during interventions or in the first days on analgesia requirement or other pain score not detailed enough to bee exploit. Three studies find similar results in favour of a pain reduction for SA [29, 30, 41]. Ho et al. find a similar pain between treatments [37]. Only Marx et al. investigated the dyspnea during or after the intervention by a score [11]. The results were non-significant with MD =-0.3 95% CI=[-0.72, 0.12] 24 h after treatment.

#### Recurrence in one year

The results regarding the recurrence of pneumothorax within one year of SA versus CTD for management of PSP was OR = 1.00 (95% CI=[0.65, 1.54], *p* = 1.00) (eight studies, I^2^ = 23%, *p* = 0.24) (Supplementary data Fig. 26). Considering only RCT studies, the results was OR = 0.84 (95% CI=[0.58, 1.23], *p* = 0.37) (six studies, I^2^ = 7%, *p* = 0.37) (Supplementary data Fig. 27). The sensitivity analysis stratified on the quality of the studies yield to the same results. The funnel plot did not reveal any publication bias (Supplementary data Fig. 28).

#### Length of hospitalization

The results regarding the length of hospitalization of SA versus CTD for management of SP was MD = -2.59 (95% CI=[-3.57, -1.61], *p* < 0.001) in favour of SA (eleven studies, I^2^ = 93%, *p* < 0.001) (Supplementary data Fig. 29). Considering only RCT studies, the results was MD=-2.56 (95% CI= [-3.85, -1.27], *p* < 0.001) in favour of SA (nine studies, I^2^ = 94%, *p* < 0.001) (Supplementary data Fig. 30). The sensitivity analysis stratified on the quality of the studies yield to the same results.

The results regarding the length of hospitalization of SA versus CTD of PSP was MD=-2.38 (95% CI=[-2.93, -1.83], *p* < 0.001) in favour of SA (height studies, I^2^ = 62%, *p* = 0.009) (Supplementary data Fig. 31). Considering only RCT studies, the results was MD=-2.17 (95% CI=[-2.83, -1.51], *p* < 0.001) in favour of SA (six studies, I^2^ = 55%, *p* = 0.05) (Supplementary data Fig. 32). The sensitivity analysis stratified on the quality of the studies yield to the same results.

Only Thelle et al. assessed the length of hospitalization in SSP [31]. They identified a significantly shorter duration of hospitalization for the SA (median = 2.5 days) than CTD (median = 5.5 days), *p* = 0.049. We observed a potential publication bias with the funnel plots diagrams analysis with many articles in favour of SA (Supplementary data Figs. 33 and 34).

## Discussion

This meta‑analysis found no difference in efficacy or recurrence between SA and CTD for the therapeutic management of SP. Considering secondary outcomes, SA appears to be better tolerated and to reduce the length of hospitalization.

Several meta-analyses have assessed the efficacy of SA versus CTD in the management of pneumothorax. A 2017 Cochrane review demonstrated a higher immediate success rate associated with CTD [10]. However, this finding has been challenged by studies conducted by Mummadi et al., Vuong et al., and Tan et al., which reported comparable efficacy between the two treatments modalities, along with a reduction in hospital length of stay associated with SA [6–8]. Their findings remain inconsistent with respect to the incidence of adverse events and recurrence rates. More recently, Cheng et al. confirmed the results of the Cochrane review by further demonstrating the effectiveness of CTD [12]. Our results differ from those previously reported, which may be explained by several factors. In comparison with the previous study, our analysis included a total of 20 articles, incorporating nine additional high-quality observational studies. This approach provides a more comprehensive and representative overview of the literature, with the inclusion of an additional 1691 subjects, thereby offering a more exhaustive assessment of the management of SP. Among the observational studies, only comparative cohort studies were included, and sensitivity analyses were performed according to study quality and design. These methodological choices likely contributed to minimizing bias, although effect estimates were derived from unadjusted event counts. In addition, efficacy judgment criteria were divided into four categories: immediately, 24 h, seven days and one-year. This approach improves the result’s precision of the estimates and reduces clinical heterogeneity. The robustness of these findings was further supported by sensitivity analyses stratified according to study quality and design, which did not materially affect the main results. We sought to improve methodological precision by clearly defining outcomes and attempting to minimize heterogeneity. Nevertheless, residual heterogeneity persisted across studies. This may be attributable to variations in pneumothorax size, outcome definitions, procedural techniques and materials used across center. Although this heterogeneity was partially addressed through the use of a random-effects model, the findings should be interpreted with caution. Likewise, we observed substantial variability in the number of SA attempts across studies. The lack of data on rescue interventions prevents a clear assessment of the efficacy of a single SA procedure independent of the effects of subsequent treatments. The observed effectiveness of SA at 24 h and seven days may be attributable to the overall SA-first management strategy rather than to the efficacy of the initial aspiration procedure alone.

In contrast to the findings reported by Cheng et al., our results are in accordance with the most recent clinical guidelines. Both the French guidelines and the British Thoracic Society guidelines recommend the used of SA for the management of symptomatic or large PSP [5, 13]. However, outpatient management using chest drainage kits (mini-CTD with one-way valve) is being developed as an alternative strategy aimed at optimizing treatment efficacy while improving patients’ quality of life during management [45, 46]. For patients presenting with small or minimally symptomatic PSP, conservative management appears to be the preferred therapeutic approach. In contrast, for SSP, CTD is recommended as a first-line treatment in order to optimize therapeutic efficacy, while preserving the possibility of subsequent chemical pleurodesis when clinically indicated [5]. It is also important to emphasize that our systematic review identified only a limited number of studies specifically investigating SSP, resulting in a relatively small sample size available for inclusion in the meta-analysis. Consequently, conclusions regarding the management of SSP should be interpreted with caution.

In healthcare settings, cost has become a key determinant in therapeutic decision-making. Although SA has not been shown to be more effective than CTD in the management of SP, health economic considerations may support its preferential use. In 2023, Eamer et al. conducted a meta-analysis comparing SA and CTD for the management of PSP, including a cost–utility analysis. The mean cost per treatment was $13,126 for SA versus $14,658 for CTD, with corresponding utility values of 0.77 and 0.79, respectively [9]. These findings are supported by a 2025 U.S. study, which demonstrated that SA is more cost-effective than CTD, with respective costs of $16,766 and $33,728 for PSP [47].

## Conclusion

This meta-analysis shows no difference in the efficacy between SA and CTD in the treatment of SP, especially for PSP. However, the additional benefits of SA over CTD in clinical patient management support prioritizing this strategy, in line with international guideline recommendations.

## Supplementary Information

Supplementary Material 1.

## Data Availability

Data sharing is not applicable to this article as no datasets were generated or analysed during the current study. The data used in this study are available in full in the studies cited to conduct this systematic review and meta-analysis.

## Abbreviations

CI: Confidence interval
CTD: Chest tube drainage
MD: Mean difference
NOS: Newcastle-Ottawa Scale
OR: Odds ratios
PSP: Primary spontaneous pneumothorax
SA: Simple aspiration
SSP: Secondary spontaneous pneumothorax
SP: Spontaneous pneumothorax
