# Supplementary material for: Should we abandon the simple aspiration in favour of chest tube drainage in the management of spontaneous pneumothorax? A systematic review and meta-analysis
Source: BMC Pulm Med. 2026 Jun 25;26:403. doi: 10.1186/s12890-026-04447-0 (PMC13555879; doi:10.1186/s12890-026-04447-0)
Supplement: Supplementary file 1 [file 12890_2026_4447_MOESM1_ESM.docx]

Supplementary data

Supplementary data Table 1: bibliographic research by database

Embase

('pneumothorax'/exp OR pneumothorax:ti,ab,kw) AND ('surgical drainage'/exp OR 'drainage/aspiration system'/exp OR 'drainage':ti,ab,kw OR 'paracentesis'/exp OR 'thoracocentesis'/exp OR paracentes*:ti,ab,kw OR centes*:ti,ab,kw OR thoracocentes*:ti,ab,kw OR exsufflation:ti,ab,kw OR aspiration:ti,ab,kw OR pleuracentes*:ti,ab,kw OR pleurocentes*:ti,ab,kw) AND ('crossover procedure'/exp OR 'double-blind procedure'/exp OR 'randomized controlled trial'/exp OR 'single-blind procedure'/exp OR random* OR factorial* OR crossover* OR (cross NEXT/1 over*) OR placebo* OR (doubl* NEAR/1 blind*) OR (singl* NEAR/1 blind*) OR assign* OR allocat* OR volunteer*)

PubMed

1	(pneumothorax[MeSH Terms]) OR (Pneumothorax[Title/Abstract])

2	(drainage[MeSH Terms]) OR (drainage[Title/Abstract])

3	((((((((paracentesis[MeSH Terms]) OR (Thoracocentesis[MeSH Terms])) OR (Paracentes*[Title/Abstract])) OR (centes*[Title/Abstract])) OR (Thoracocentes*[Title/Abstract])) OR (exsufflation[Title/Abstract])) OR (aspiration[Title/Abstract])) OR (pleuracentes*[Title/Abstract])) OR (pleurocentes*[Title/Abstract])

4	#1 AND (#2 OR #3)

Central

#1	MeSH descriptor: [Pneumothorax] explode all trees	614

#2	Pneumothorax	2996

#3	#1 OR #2	2996

#4	MeSH descriptor: [Drainage] explode all trees	3616

#5	drainage	11936

#6	#4 OR #5	13227

#7	MeSH descriptor: [Paracentesis] explode all trees	436

#8	MeSH descriptor: [Thoracentesis] explode all trees	21

#9	paracentes* OR centes* OR thoracocentes* OR exsufflation OR aspiration OR pleuracentes* OR pleurocentes*	12474

#10	#7 OR #8 OR #9	12709

#11	#3 AND (#6 OR #10)	691

Supplementary data Table 2: Population-Intervention-Comparison-Outcome-Study design (PICOS) statement.

| Population (P) | adults having presented a SP managed by thoracic tube drainage or simple aspiration |
| --- | --- |
| Intervention (I) | simple aspiration |
| Comparison (C) | thoracic tube drainage |
| Outcome (O) | immediate efficiency of the treatments, efficiency of interventions in 24 hours and in seven days, recurrence rate in one year, length of hospital stays, occurrence of adverse effect and tolerance of treatment |
| Study design (S) | Randomized control trials and observational studies |

Supplementary data Figure 1: Meta-analysis of immediate success with RCT for all the SP.

Supplementary data Figure 2: Meta-analysis of immediate success with all studies for primary SP.

Supplementary data Figure 3: Meta-analysis of immediate success with RCT for primary SP.

Supplementary data Figure 4: Meta-analysis of immediate success with all studies for secondary SP.

Supplementary data Figure 5: funnel plot of immediate success for all the SP between simple aspiration and chest tube drainage

Supplementary data Figure 6: funnel plot of immediate success for primary SP between simple aspiration and chest tube drainage

Supplementary data Figure 7: funnel plot of immediate success for secondary SP between simple aspiration and chest tube drainage

Supplementary data Figure 8: Meta-analysis of success in 24 hours with RCT for primary SP.

Supplementary data Figure 9: funnel plot of success in 24h for SP between simple aspiration and chest tube drainage

Supplementary data Figure 10: funnel plot of success in 24h for primary SP between simple aspiration and chest tube drainage

Supplementary data Figure 11: Meta-analysis of success in seven days with RCT for all the SP.

Supplementary data Figure 12: Meta-analysis of success in seven days with all studies for primary SP.

Supplementary data Figure 13: Meta-analysis of success in seven days with RCT for primary SP.

Supplementary data Figure 14: Meta-analysis of success in seven days with RCT for secondary SP.

Supplementary data Figure 15: funnel plot of success in 7 days for SP between simple aspiration and chest tube drainage

Supplementary data Figure 16: funnel plot of success in 7 days for primary SP between simple aspiration and chest tube drainage

Supplementary data Figure 17: funnel plot of success in 7 days for secondary SP between simple aspiration and chest tube drainage

Supplementary data Figure 18: Meta-analysis of adverse events with all studies for all the SP.

Supplementary data Figure 19: Meta-analysis of adverse events with RCT for all the SP.

Supplementary data Figure 20: Meta-analysis of adverse events with all studies for primary SP.

Supplementary data Figure 21: Meta-analysis of adverse events with RCT for primary SP.

Supplementary data Figure 22: funnel plot of occurrence of adverse events for SP between simple aspiration and chest tube drainage

Supplementary data Figure 23: funnel plot of occurrence of adverse events for primary between simple aspiration and chest tube drainage

Supplementary data Figure 24: Meta-analysis of pain with RCT for primary SP.

Supplementary data Figure 25: funnel plot of tolerance of treatment for primary SP between simple aspiration and chest tube drainage

Supplementary data Figure 26: Meta-analysis of recurrence in one year with all studies for primary SP.

Supplementary data Figure 27: Meta-analysis of recurrence in one year with RCT for primary SP.

Supplementary data Figure 28: funnel plot of recurrence in one year for primary SP between simple aspiration and chest tube drainage

Supplementary data Figure 29: Meta-analysis of length of hospitalization with all articles for all SP.

Supplementary data Figure 30: Meta-analysis of length of hospitalization with RCT for all SP.

Supplementary data Figure 31: Meta-analysis of length of hospitalization with all articles for PSP.

Supplementary data Figure 32: Meta-analysis of length of hospitalization with RCT for PSP.

Supplementary data Figure 33: funnel plot of length of hospitalization for SP between simple aspiration and chest tube drainage

Supplementary data Figure 34: funnel plot of length of hospitalization for primary SP between simple aspiration and chest tube drainage
